# How to Fix a Bad Trip? Hallucinogen Persistent Perception Disorder Treatment in Review

**DOI:** 10.1192/bjo.2026.11219

**Published:** 2026-06-30

**Authors:** Aleeha Batool, Chaitanya Morey

**Affiliations:** Hampshire and Isle of Wight Healthcare NHS Foundation Trust, Southampton, United Kingdom

## Abstract

**Aims:**

Hallucinogen Persistent Perception Disorder (HPPD) is characterised by a recurrence of hallucinogen-induced perceptual symptoms after the cessation of hallucinogen use, leading to clinically significant impairment in functioning. It remains difficult to diagnose due to multiple comorbid diagnoses and poor understanding in psychiatry. For these reasons documented incidence also remains low. There are currently no established guidelines on treatment of HPPD which is a major gap in existing literature. This review aims to assimilate current evidence on treatment of HPPD, and suggest avenues for further systematised research into this complex disease.

**Methods:**

Our inclusion criteria was any review article, meta-analysis, randomised control trial, case report, and letter to the editor on treatment of HPPD published in English until December 2025. Research databases PubMed and GoogleScholar were searched using the key words “Hallucinogen Persistent Perception Disorder” yielding 111 articles. These were then screened to meeting inclusion criteria and relevance to aim. 34 studies were included in our final review.

**Results:**

Our review of studies on HPPD treatment found that several (n=9) advocate for α2 adrenergic agonists (particularly clonidine) orbenzodiazepines (particularly clonazepam) to be effective. These target the distress and anxiety associated with HPPD. The perceptual symptoms of HPPD have shown good response to anti-epileptic drugs (particularly lamotrigine) in case reports (n=5). Studies (n=5) on treatment with antipsychotic agents counterintuitively demonstrated poor response and worsening of perceptual symptoms. Two case reports (n=2) demonstrated improvement on combination therapy (olanzapine with fluoxetine and risperidone with escitalopram) however selective serotonin reuptake inhibitors alone (n=1) have not shown improvement.

Anecdotal treatments in literature include conservative measures, psychotherapy, eye movement desensitisation and reprocessing, onabotulinum toxinA, and naltrexone.

Neuromodulation has been successful at treating HPPD in two case reports using transcranial direct current stimulation and repetitive transcranial magnetic stimulation, with sustained responses at 4 weeks and 4 months respectively. More robust studies with longer term follow-up are needed.

**Conclusion:**

Treatment modalities recommended for HPPD have a weak evidence base. The ambiguity of HPPD presentation, frequent comorbid conditions influencing pharmacological choice and variability in response to treatments make it difficult to draw large-scale conclusions from these results. Larger scale and case-controlled studies remain a challenge owing to diagnostic and ethical considerations. We propose that, given the safety and efficacy of neuromodulation, this should be explored further. Additionally, more research needs to be done for pharmacological treatments to confirm efficacy.

